# Association between serum β2‐microglobulin and left ventricular hypertrophy in patients with type 2 diabetes mellitus: A cross‐sectional study

**DOI:** 10.1111/1753-0407.13599

**Published:** 2024-08-19

**Authors:** Yuling Zhang, Guiliang Peng, Weiling Leng, Ying Li, Haiyan Li, Ling Zhou, Lichao Ge, Jiaqing Shao, Xing Li, Min Long

**Affiliations:** ^1^ Department of Endocrinology Southwest Hospital, Army Medical University (Third Military Medical University) Chongqing China; ^2^ Center for Medical Big Data and Artificial Intelligence, Southwest Hospital, Army Medical University (Third Military Medical University) Chongqing China; ^3^ Department of Ultrasonography Chenjiaqiao Hospital Chongqing China; ^4^ Department of Endocrinology Jinling Hospital, Nanjing Medical University Nanjing China; ^5^ Department of Endocrinology Jinling Hospital, Affiliated Hospital of Medical School, Nanjing University Nanjing China; ^6^ Department of Endocrinology Jinling Hospital, School of Medicine, Southeast University Nanjing China

**Keywords:** Beta 2‐microglobulin, left ventricular hypertrophy, type 2 diabetes mellitus

## Abstract

**Background:**

Beta 2‐microglobulin (β2‐MG) is a component of the class I major histocompatibility complex (MHCI) and has recently been reported to be involved in type 2 diabetes mellitus (T2DM) and cardiovascular disease. However, the association of β2‐MG with left ventricular hypertrophy (LVH) in T2DM patients remains unknown. This study aims to investigate the correlation between serum β2‐MG and LVH in T2DM patients.

**Methods:**

The retrospective analysis included 4602 eligible T2DM patients, divided into LVH and non‐LVH groups based on echocardiography results. Serum β2‐MG levels were measured, and participants were categorized into four groups (Q1–Q4) by their serum β2‐MG quartile. The relationship of serum β2‐MG level with LVH was evaluated using logistic regression, restricted cubic spline (RCS), subgroup analysis, and machine learning.

**Results:**

The prevalence of LVH in T2DM patients was 31.12%. Each standard deviation increase in serum β2‐MG level corresponded to a 1.17‐fold increase in the prevalence of LVH [OR = 1.17, (95% CI: 1.05–1.31); *p* = 0.006]. When considering β2‐MG as a categorical variable (quartile), Q3 [OR = 1.36, (95% CI: 1.09–1.69); *p* = 0.007] and Q4 [OR = 1.77, (95% CI: 1.36–2.31); *p* < 0.001] had a significantly higher prevalence of LVH than Q1. RCS analysis found a nonlinear association between β2‐MG and LVH prevalence (*p* for nonlinearity <0.05). Additionally, machine learning results confirmed the importance of β2‐MG for LVH in T2DM patients.

**Conclusion:**

Elevated serum β2‐MG levels were likely to be associated with an increased prevalence of LVH in T2DM patients, suggesting its potential role in LVH development.

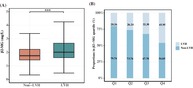

## Graphical abstract

Highlights

- Serum β2‐MG significantly increased in T2DM patients with left ventricular hypertrophy (LVH).
- Serum β2‐MG levels were associated with LVH prevalence in patients with T2DM.

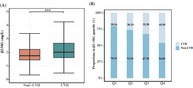

## 1 INTRODUCTION

The increasing prevalence of type 2 diabetes mellitus (T2DM) is a global public health challenge.^1^ It is well known that cardiovascular disease (CVD) significantly increases the risk of mortality in patients with T2DM.^2^ Left ventricular hypertrophy (LVH) has been established as a reliable predictor of cardiovascular events and mortality, and is common in T2DM, with a prevalence of 32%–71%.^3^ It is a cardiac structural abnormality considered characteristic of diabetic cardiomyopathy.^4^, ^5^ T2DM increases the risk of left heart dysfunction, including increased left ventricular mass and wall thickness and impaired diastole and systole,^6^ which may be linked to inflammation, oxidative stress, insulin resistance, glycosylation, and renal function.^3^ Previous studies have shown that metformin,^7^ sodium‐glucose cotransporter 2 (SGLT2) inhibitors,^8^ and allopurinol^9^ attenuate cardiovascular events and mortality in T2DM in part by promoting LVH regression and reversing left ventricular remodeling, but the exact mechanisms of these effects are yet to be fully understood. Therefore, studies of LVH in T2DM may help in finding ways to reduce cardiovascular events and prevent early mortality in this patient population.

Beta 2‐microglobulin (β2‐MG) is a low molecular weight protein (11.8 kD) found in the cell membrane of lymphocytes and platelets. It is the beta light‐chain component of the major class I histocompatibility complex (MHC‐I). The β2‐MG participates in immune activation, inflammatory states, and stress responses.^10^ Serum β2‐MG is an early marker of renal function, as approximately 99% is reabsorbed by proximal renal tubules and completely metabolized. Recent evidence supports a strong association between elevated serum β2‐MG and various cardiovascular diseases, including cardiac valve calcification,^11^ peripheral arterial disease,^12^ coronary heart disease,^13^ atrial fibrillation,^14^ and heart failure.^15^ Elevated serum β2‐MG has also been positively correlated with all‐cause and cardiovascular mortality in the general population^16^, ^17^ and T2DM patients with normal renal function.^18^

However, the association of serum β2‐MG with the development of LVH in T2DM patients has not been explored. The objective of this study was to evaluate the correlation of change in serum β2‐MG and LVH in T2DM patients.

## 2 PATIENTS AND METHODS

### 2.1 Participants

This cross‐sectional study was approved (KY2024007) by the Southwest Hospital Human Research Ethics Committee. Given the retrospective nature of this study and the anonymity of participant data, the institutional reviewer waived the requirement for informed consent. T2DM patients who were hospitalized at the Department of Endocrinology at Southwest Hospital between 2018 and 2023 were enrolled. The diagnosis of T2DM was consistent with the criteria established by the American Diabetes Association (2020).^19^ Patients (1) younger than 18 years of age; (2) pregnant; (3) with acute complications of diabetes mellitus; (4) without complete β2‐MG and cardiac ultrasound data; (5) with severe valvular heart disease, atrial fibrillation, renal diseases (excluding diabetic nephropathy), end‐stage renal disease, stroke, infective diseases, hematological, or other malignant diseases were excluded. The study ultimately included a total of 4602 patients with T2DM who met the inclusion criteria (A flowchart is provided in Figure S1).

### 2.2 Clinical parameters

Baseline clinical data of the patients were obtained from their electronic medical records, including age, sex, height, weight, systolic blood pressure (SBP), diastolic blood pressure (DBP), drinking history, smoking history, hypertension history, hyperlipidemia history, coronary heart disease (CHD) history, heart failure (HF) history, and diabetes complications. The body mass index (BMI) was calculated as body weight in kilograms/height in meters squared (kg/m^2^) and body surface area (BSA) was calculated using the formula: 0.0061 × height (cm) + 0.0125 × weight (kg)−0.1529.

Laboratory parameters included fasting blood glucose (FBG), glycated hemoglobin (HbA1c), total cholesterol (TC), triglycerides (TG), high‐density lipoprotein cholesterol (HDL‐C), low‐density lipoprotein cholesterol (LDL‐C), alanine aminotransferase (ALT), aspartate aminotransferase (AST), uric acid (UA), estimated glomerular filtration rate (eGFR), β2‐MG, C‐reactive protein (CRP) and interleukin‐6 (IL‐6), and N‐terminal pro brain natriuretic peptide (NT‐proBNP). All blood samples were collected after an overnight fast of at least 8 h and were tested immediately after being obtained. The test methods and equipment for the clinical indicators are detailed in Table S5.

### 2.3 Ultrasound examination

Echocardiography was performed with participants in a supine position and in a calm state by a certified sonographer using a high‐resolution ultrasound system (Philips IE33, Philips, USA). Left ventricular posterior wall thickness (LVPWT), interventricular septal thickness (IVST), left ventricular end diastolic diameter (LVDd), left ventricular end diastolic, and end systolic diameters were obtained using the M‐mode. The left ventricular ejection fraction (LVEF) was calculated. Early (E) and late (A) diastolic peak mitral velocities were assessed using pulsed wave Doppler echocardiography, and the peak early diastolic velocity of the septal and lateral mitral annulus (e') was measured using pulsed tissue Doppler ultrasound. Left ventricular myocardial mass (LVM) and left ventricular myocardial mass index (LVMI) were calculated with the Devereux formula. LVM (g) = 0.8 × 1.04 × [(IVST + LVPWT + LVDd)^3^ × LVD^3^] + 0.6 and LVMI (g/m^2^) = LVM/BSA (upper reference limit for normal LVMI: 95 g/m^2^ for women, 115 g/m^2^ for men). LVH was defined as an LVMI greater than the upper reference limit.^20^, ^21^

### 2.4 Machine learning

To investigate the association between the β2‐MG level and LVH in these T2DM patients, we used a machine learning method based on a random forest model for feature selection of variables to assess the importance of serum β2‐MG for LVH occurrence. Shapley additive explanations (SHAP) analysis was used to explain the decision‐making process of the machine learning model, including sorting the features by importance, showing the association between observed values and prevalence, and determining cutoff values. When the SHAP value of feature observation was greater than zero, its effect on model output was positive. This method helps to increase the visibility and interpretability of the machine learning model.

### 2.5 Statistical analysis

Normally distributed continuous variables were reported as means ± standard deviation (SD), and independent sample *t*‐tests were used to compare between‐group differences. Non‐normally distributed continuous variables were reported as medians, with the interquartile range (25%–75%) used for description. Group comparisons were performed using Mann–Whitney U tests. Categorical variables were reported as frequencies and percentages, and multiple‐group comparisons used *χ* ^2^ tests. Pearson's correlation analysis was used to investigate the association between serum β2‐MG and cardiac‐related indicators in individuals with T2DM. Multivariate logistic regression was performed to assess the independent association of β2‐MG with LVH occurrence in these T2DM patients. Analysis was adjusted for potentially confounding variables including age, sex, BMI, smoking, drinking, SBP, DBP, hypertension, hyperlipidemia, DN, CHD, HF, antihypertensive drugs, lipid‐lowering drugs, antidiabetes drugs, insulin, FBG, HbA1c, TC, TG, HDL‐C, LDL‐C, UA, eGFR, ALT, AST, CRP, IL‐6, and NT‐proBNP. Multivariate‐adjusted restricted cubic spline (RCS) regression was performed to investigate the dose–response association between serum β2‐MG levels and LVH. Subgroup analyses were conducted to further evaluate the stability and reliability of the main results. The statistical analysis and graphing were performed by RStudio (version 2023.12.0 + 369). Machine learning model development and explanation were conducted in Python, version 3.11.5, scikit‐learn, version 1.3.0, and SHAP, version 0.44.0. The missing data accounted for 2.99% of the total data, and the missing data were imputed by random forest method. Statistical significance was defined as a *p*‐value <0.05.

## 3 RESULTS

### 3.1 Baseline characteristics of the study patients

**FIGURE 1:**
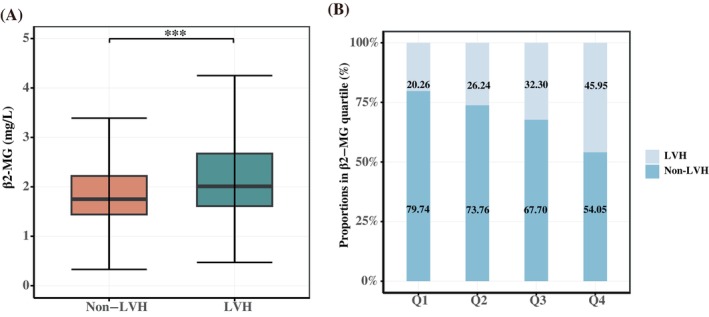
The β2‐MG level and β2‐MG quantile proportions in LVH and non‐LVH patients with T2DM. (A) Comparison of β2‐MG level between LVH group and non‐LVH group (***p < 0.001). (B) Prevalence of LVH in different β2‐MG quartiles (Q1–Q4). LVH, left ventricular hypertrophy.

As shown in Table S1, the study included 4602 eligible T2DM patients with a median age of 59 years, 61.15% were men, and the prevalence of LVH was 31.12%. The LVH group patients were older, had higher SBP, CRP, interleukin (IL‐6), and NT‐proBNP levels, and lower ALT, AST, UA, and eGFR levels than the non‐LVH group patients. Patients in the LVH group had a higher incidence of hypertension, diabetic nephropathy (DN), CHD, and HF than those in the non‐LVH group (*p* < 0.05). In addition, patients with LVH had higher β2‐MG levels than those without LVH (Figure 1A). Information on the use of antidiabetes drug during hospitalization is shown in Table S2.

### 3.2 Comparison of clinical characteristics among the β2‐MG quartile groups

The patients were assigned to study groups defined by the serum β2‐MG quartile (Q1–Q4). As shown in Table 1, participants in the highest β2‐MG quartile were likely to be older, hypertensive, and have DN or HF compared with those in the lowest quartile (*p* < 0.05). As shown in Table 2, UA, CRP, IL‐6, and NT‐proBNP were increased in patients with high β2‐MG levels and glucolipid metabolism indicators (FBG, TC, LDL‐C, and HDL‐C) and eGFR were decreased. As for echocardiographic parameters, compared with the lowest quartile group (Q1), patients in the highest quartile (Q4) had increased IVST, LVDd, LVPWT, and LVM and decreased left ventricle systolic and diastolic function indicators, such as LVEF and E/A ratio decline (all *p* < 0.05). The occurrence of LVH increased as serum β2‐MG level increased, with 236 patients in Q1 (20.26%), 306 (26.24%) in Q2, 363 (32.30%) in Q3, and 527 (45.95%) in Q4 (Figure 1B).

**TABLE 1:** Baseline characteristics according to β2‐MG quartile.

Characteristic	Q1 (*n* = 1165)	Q2 (*n* = 1166)	Q3 (*n* = 1124)	Q4 (*n* = 1147)	*p*‐value
β2‐MG, mg/L	<1.50	1.50–1.88	1.88–2.53	>2.53	‐
Age, years	54.00 [45.00, 60.00]	59.00 [52.00, 65.00]	60.00 [55.00, 68.00]	60.00 [55.00, 68.00]	<0.001
Male sex	683 (58.63)	728 (62.44)	695 (61.8)	708 (61.73)	0.229
SBP, mmHg	126.00 [116.00, 136.00]	128.00 [119.00, 138.00]	129.00 [120.00, 141.00]	134.00 [121.00, 148.00]	<0.001
DBP, mmHg	81.00 [75.00, 89.00]	82.00 [75.00, 88.00]	81.00 [74.00, 88.00]	81.00 [74.00, 90.00]	0.554
Smoking	439 (37.68)	480 (41.17)	456 (40.57)	519 (45.25)	0.003
Drinking	456 (39.14)	460 (39.45)	421 (37.46)	456 (39.76)	0.680
BMI, kg/m^2^	24.27 [22.14, 26.49]	24.69 [22.56, 26.82]	24.79 [22.59, 26.82]	24.36 [22.14, 26.81]	0.016
Hypertension	607 (52.10)	756 (64.84)	785 (69.84)	887 (77.33)	<0.001
Hyperlipidemia	318 (27.30)	349 (29.93)	282 (25.09)	208 (18.13)	<0.001
DN	346 (29.70)	412 (35.33)	512 (45.55)	840 (73.23)	<0.001
CHD	186 (15.97)	259 (22.21)	263 (23.40)	258 (22.49)	<0.001
HF	3 (0.26)	8 (0.69)	19 (1.69)	63 (5.49)	<0.001
Antihypertensive drugs	156 (13.39)	219 (18.78)	214 (19.04)	271 (23.63)	<0.001
Lipid‐lowering drugs	278 (23.86)	325 (27.87)	273 (24.29)	264 (23.02)	0.036
Antidiabetes drugs	325 (27.90)	373 (31.99)	304 (27.05)	277 (24.15)	<0.001
Insulin	280 (24.03)	281 (24.10)	247 (21.98)	279 (24.32)	0.52

*Note*: Values are presented as number (%), mean ± standard deviation, or median (interquartile range).

Abbreviations: BMI, body mass index; CHD, coronary heart disease; DBP, diastolic blood pressure; DN, diabetic nephropathy; HF, heart failure; SBP, systolic blood pressure.

**TABLE 2:** Baseline laboratory and echocardiographic characteristics according to β2‐MG quartile.

Characteristic	Q1 (*n* = 1165)	Q2 (*n* = 1166)	Q3 (*n* = 1124)	Q4 (*n* = 1147)	*p*‐value
Laboratory parameters					
FBG, mmol/L	9.50 [7.54, 11.80]	8.91 [7.17, 11.24]	8.91 [7.38, 11.07]	8.86 [7.10, 11.11]	<0.001
HbA1c, %	8.55 [7.20, 10.37]	8.15 [7.09, 9.80]	8.31 [7.21, 9.90]	8.44 [7.30, 10.00]	0.006
TC, mmol/L	4.82 [4.12, 5.54]	4.69 [4.00, 5.37]	4.64 [3.93, 5.35]	4.47 [3.76, 5.37]	<0.001
LDL‐C, mmol/L	2.93 [2.45, 3.45]	2.88 [2.41, 3.37]	2.86 [2.40, 3.31]	2.77 [2.30, 3.39]	<0.001
TG, mmol/L	1.74 [1.17, 2.77]	1.69 [1.18, 2.64]	1.71 [1.24, 2.68]	1.75 [1.27, 2.51]	0.730
HDL‐C, mmol/L	1.16 [0.99, 1.35]	1.13 [0.97, 1.31]	1.11 [0.96, 1.28]	1.06 [0.90, 1.27]	<0.001
AST, IU/L	21.56 [17.68, 27.75]	22.40 [18.67, 28.36]	22.62 [18.80, 29.48]	21.87 [17.61, 28.23]	<0.001
ALT, IU/L	23.60 [16.60, 33.26]	24.14 [17.43, 34.70]	23.30 [17.07, 34.77]	19.90 [14.15, 29.51]	<0.001
UA, μmol/L	310.00 [258.00, 370.00]	323.43 [272.51, 379.98]	340.41 [282.00, 400.50]	375.24 [312.92, 443.01]	<0.001
eGFR, ml/min/1.73 m^2^	112.84 [103.33, 126.99]	103.37 [94.02, 113.09]	93.38 [81.86, 104.88]	64.77 [41.67, 87.91]	<0.001
CRP, mg/L	2.21 [0.96, 7.76]	2.44 [1.00, 8.74]	3.38 [1.35, 11.86]	7.35 [1.98, 32.80]	<0.001
IL‐6, ng/L	4.11 [1.50, 10.39]	4.35 [1.86, 9.67]	5.40 [2.04, 13.74]	9.29 [3.57, 24.74]	<0.001
NT‐proBNP, pg/mL	43.07 [20.25, 84.60]	46.80 [20.92, 101.79]	66.40 [31.40, 142.74]	256.15 [83.40, 1114.05]	<0.001
Echocardiographic parameters					
IVST, cm	0.99 [0.89, 1.10]	1.02 [0.92, 1.13]	1.04 [0.93, 1.16]	1.08 [0.96, 1.23]	<0.001
LVDd, cm	4.60 [4.30, 4.80]	4.60 [4.40, 4.80]	4.60 [4.40, 4.90]	4.70 [4.40, 5.00]	<0.001
LVPWT, cm	0.91 [0.84, 0.98]	0.93 [0.85, 1.01]	0.94 [0.85, 1.02]	0.97 [0.88, 1.07]	<0.001
LVM, g	146.95 [125.97, 172.49]	154.20 [131.99, 179.61]	158.81 [134.88, 184.44]	167.45 [144.89, 201.72]	<0.001
LVEF, %	64.00 [61.00, 68.00]	64.00 [60.00, 67.00]	64.00 [60.00, 67.00]	63.00 [59.00, 67.00]	<0.001
E, cm/s	65.00 [55.00, 77.00]	66.00 [57.00, 77.00]	66.00 [57.00, 76.00]	65.00 [54.50, 76.00]	0.020
A, cm/s	78.00 [68.00, 91.00]	85.00 [74.00, 96.00]	87.00 [75.00, 100.00]	91.00 [79.00, 103.50]	<0.001
E/A ratio	0.81 [0.68, 1.00]	0.77 [0.66, 0.90]	0.76 [0.64, 0.88]	0.71 [0.59, 0.84]	<0.001
Septal e, cm/s	7.00 [6.00, 8.00]	6.70 [6.00, 8.00]	6.50 [5.70, 7.40]	6.00 [5.30, 7.10]	<0.001
Lateral e, cm/s	9.46 [8.74, 10.90]	9.13 [8.49, 10.05]	8.91 [8.30, 9.66]	8.55 [8.00, 9.34]	<0.001
e', cm/s	8.29 [7.49, 9.50]	7.97 [7.26, 8.99]	7.71 [7.06, 8.50]	7.47 [6.87, 8.12]	<0.001

*Note*: Values are presented as number (%), mean ± standard deviation, or median (interquartile range).

Abbreviations: A, trans‐mitral late diastolic peak velocity; ALT, alanine aminotransferase; AST, aspartate aminotransferase; CRP, C‐reactive protein; e', (Septal e + Lateral e)/2; E, trans‐mitral early diastolic peak velocity; eGFR, estimated glomerular filtration rate; FBG, fasting blood glucose; HbA1c, glycated hemoglobin; HDL‐C, high‐density lipoprotein cholesterol; IL‐6, interleukin 6; IVST, interventricular septal thickness; LDL‐C, low‐density lipoprotein cholesterol; LVDd, left ventricular end diastolic dimension; LVEF, left ventricular ejection fraction; LVM, left ventricular mass; LVPWT, left ventricular posterior wall thickness at end‐diastole; NT‐proBNP, N‐terminal pro brain natriuretic peptide; TC, total cholesterol; TG, triglycerides; UA, uric acid; β2‐MG, β2‐microglobulin.

### 3.3 Correlation of serum β2‐MG level and cardiac structural and functional parameters

Pearson's correlation analysis revealed a positive correlation between serum β2‐MG level and left ventricular structural parameters (IVST, LVDd, LVPWT, and LVM) and a negative correlation with left ventricular systolic and diastolic function‐related indicators such as LVEF and E/A ratio (*p* < 0.05). In addition, serum β2‐MG level was positively correlated with SBP, CRP, IL‐6, NT‐proBNP, and negatively correlated with eGFR level (*p* < 0.05) (Table S3).

### 3.4 Independent association between serum β2‐MG level and the prevalence of LVH

Multivariate logistic regression analysis was used to investigate the independent correlation between serum β2‐MG level and the prevalence of LVH in T2DM patients. After adjusting for the confounding factors in model 3, each SD increase in serum β2‐MG corresponded to a 17% increase in the prevalence of LVH [OR = 1.17, (95% CI: 1.05–1.31), *p* = 0.006]. When β2‐MG was considered a categorical variable (quartile), the prevalence of LVH was significantly higher in patients in Q1 than in those in Q3 [OR = 1.36, (95% CI: 1.09–1.69); *p* = 0.007] and Q4 [OR = 1.77, (95% CI: 1.36–2.31); *p* < 0.001]. The prevalence of LVH was observed to progressively escalate with higher β2‐MG levels (*p* for trend <0.001) (Table 3).

**TABLE 3:** Association between serum β2‐MG level and the prevalence of LVH in T2DM.

β2‐MG	LVH					
	Model 1 OR (95% CI)	*p‐*value	Model 2 OR (95% CI)	*p‐*value	Model 3 OR (95%CI)	*p‐*value
Continuous (per 1 SD increase)	1.67 (1.52, 1.85)	<0.001	1.38 (1.25, 1.52)	<0.001	1.17 (1.05, 1.31)	**0.006**
Quartiles (Q1–Q4)						
Q1	Reference		Reference		Reference	
Q2	1.40 (1.15, 1.70)	<0.001	1.23 (1.00, 1.52)	0.055	1.20 (0.97, 1.49)	0.094
Q3	1.88 (1.55, 2.27)	<0.001	1.45 (1.17, 1.79)	<0.001	1.36 (1.09, 1.69)	**0.007**
Q4	3.35 (2.79, 4.03)	<0.001	2.29 (1.84, 2.84)	<0.001	1.77 (1.36, 2.31)	**<0.001**
*p* for trend	<0.001		<0.001		<0.001	

*Note*: Model 1: unadjusted; Model 2: adjusted for age, sex, BMI, smoking, drinking, SBP, DBP, hypertension, hyperlipidemia, DN, CHD, HF; Model 3: adjusted for age, sex, BMI, smoking, drinking, SBP, DBP, hypertension, hyperlipidemia, DN, CHD, HF, antihypertensive drugs, lipid‐lowering drugs, antidiabetes drugs, insulin, FBG, HbA1c, TC, TG, HDL‐C, LDL‐C, UA, eGFR, ALT, AST, CRP, IL‐6, NT‐proBNP. A *p*‐value < 0.05 was considered statistically significant, and it is presented in bold.

Abbreviations: LVH, Left ventricular hypertrophy; SD, standard deviation.

**FIGURE 2:**
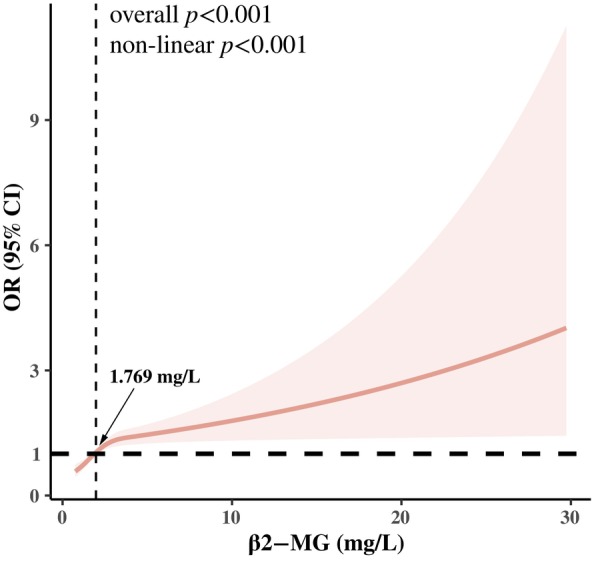
Dose–response curves of serum β2‐MG level and prevalence of LVH in T2DM patients. LVH, left ventricular hypertrophy; T2DM, type 2 diabetes mellitus.

In addition, the RCS regression model found a significant nonlinear relationship between serum β2‐MG level and LVH prevalence in these patients (*p* for nonlinear <0.05), as shown in Figure 2. It was also observed that as the β2‐MG level increased beyond 1.769 mg/L, the prevalence of LVH gradually increased.

### 3.5 Subgroup analysis

To further investigate the association between serum β2‐MG level and LVH, we conducted subgroup analyses stratified by age, sex, BMI, hypertension history, hyperlipidemia history, DN history, and CHD history. Stratified and interaction analyses showed that the association between β2‐MG level and prevalence of LVH varied with age, hypertension history, and DN (*p* for interaction <0.05). The association between β2‐MG level and LVH was stronger in patients younger than 60 years of age and in patients without hypertension and a history of DN. In addition, trends in subgroup analyses according to sex, BMI level, and hyperlipidemia status were consistent with the main study findings (Table S4).

### 3.6 Machine learning detecting the importance of serum β2‐MG for LVH in T2DM

**FIGURE 3:**
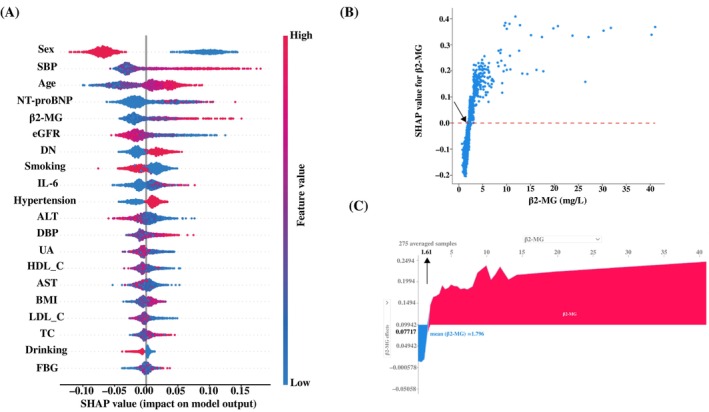
Predictive value of serum β2‐MG in LVH in T2DM assessed by machine learning. (A) SHAP summary plot of the top 20 clinical features in the random forest model. Each row represents a feature, and the horizontal coordinate represents the corresponding SHAP value. Each dot represents a patient. The redder the color, the higher the feature value, and the bluer the color, the lower its value. (B) SHAP dependence plots and (C) force plots show how β2‐MG influenced model output. β2‐MG level had a positive impact on model output. Arrows indicate the 1.61 mg/L threshold. ALT, alanine aminotransferase; AST, aspartate aminotransferase; BMI, body mass index; DBP, diastolic blood pressure; DN, diabetic nephropathy; eGFR, estimated glomerular filtration rate; FBG, fasting blood glucose; HDL‐C, high‐density lipoprotein cholesterol; IL‐6, interleukin‐6; LDL‐C, low‐density lipoprotein cholesterol; NT‐proBNP, N‐terminal pro brain natriuretic peptide; SBP, systolic blood pressure; SHAP, SHapley Additive exPlanations; TC, total cholesterol; UA, uric acid; β2‐MG, β2‐microglobulin.

A random forest machine learning algorithm was used to determine the clinical importance of serum β2‐MG for LVH in T2DM patients. SHAP analysis was used to interpret the random forest model by calculating the contribution of each variable to the prediction. The SHAP summary plot (Figure 3A) shows the contributions of the top 20 features in the model are arranged in descending order by SHAP value on the X‐axis. The X‐axis position of each point represents the SHAP value of its corresponding peer feature. The graph shows that the β2‐MG level has a positive impact on the model output, with an increase in β2‐MG level leading to an increase in SHAP value, indicating an elevated prevalence of LVH in T2DM patients. The SHAP dependence plots illustrate the association between serum β2‐MG level and the prevalence of LVH (Figure 3B). Serum β2‐MG values are on the X‐axis and the corresponding SHAP values are on the Y‐axis. A SHAP value >0 indicates an increased likelihood of LVH prevalence. There was an overall trend of an increase in LVH prevalence with increased serum β2‐MG. Moreover, force plots (Figure 3C) were employed to readily identify the high‐risk cutoff points of serum β2‐MG levels for LVH occurrence, with a determined threshold at about 1.61 mg/L.

## 4 DISCUSSION

LVH is associated with the risk of CVD and cardiovascular events and mortality by affecting ventricular function, coronary circulation, and arrhythmias. LVH is common in T2DM patients, and several studies have shown that a decrease in LVH reduced the risk of cardiovascular events and mortality in T2DM patients.^22^, ^23^ Therefore, early screening and targeted treatment of LVH are anticipated to mitigate the risk of cardiovascular events and mortality in T2DM. Recently, biomarkers have emerged as a cost‐effective tool for early screening and as potential therapeutic targets in various fields. However, only a few biomarkers (e.g., NT‐proBNP, high‐sensitivity troponin, CRP, and IL‐6) are currently used to aid in clinical decision‐making for LVH.^24^ This is the first study to investigate the relationship between serum β2‐MG level and LVH in T2DM patients. We found that higher serum β2‐MG levels were independently associated with an increased prevalence of LVH after adjusting for multiple confounding factors, and this association remains robust. This indicates that serum β2‐MG may participate in the development of LVH in T2DM patients.

The importance of serum β2‐MG in cardiovascular‐related diseases is increasingly recognized. Some studies have shown that high levels of serum β2‐MG were associated with cardiovascular events and mortality in various patient populations, including uremia,^25^ chronic kidney disease,^26^ asymptomatic carotid atherosclerosis,^27^ and the general population.^16^, ^28^ Studies have also found that serum β2‐MG levels are positively correlated with the severity of coronary artery disease^13^ and HF.^29^ These studies have indicated an association between β2‐MG and cardiovascular disorders. This study found that β2‐MG level had a significant positive correlation with age, SBP, NT‐proBNP, and UA levels and a significant negative correlation with LVEF and E/A. The association of β2‐MG with poor prognosis may be explained by the presence of cardiovascular risk factors. In addition, elevated serum β2‐MG levels have been associated with an increased risk of diabetes complications, including subclinical atherosclerosis, DN, and diabetic retinopathy.^18^ In this study, we observed that serum β2‐MG level had significant positive correlations with IVST, LVDd, LVPWT, and LVM, which are indicators of left ventricular structure. With each increase of one SD in serum β2‐MG, the prevalence of LVH increased by 17%. Moreover, the prevalence of LVH was significantly higher in the highest serum β2‐MG quartile compared with the lowest quartile. These results suggest that β2‐MG level may be associated with abnormal cardiac structural changes in patients with diabetic cardiomyopathy, and may be an effective biomarker of diseases related to diabetic cardiomyopathy.

The exact biological mechanism of serum β2‐MG in the development of LVH in T2DM is still unclear. Increasing evidence indicates that inflammation and oxidative stress participate in mediating the development of LVH.^3^, ^30^ Serum β2‐MG initiates inflammatory responses and triggers inflammatory processes, such as stimulating monocytes to secrete high levels of pro‐inflammatory cytokines including IL‐6, tumor necrosis factor, and others.^31^ In this study, the levels of the inflammatory markers IL‐6 and CRP levels were increased in the LVH group compared with the non‐LVH group. Correlation analysis revealed that serum β2‐MG levels were positively correlated with inflammatory markers. Therefore, it is reasonable to hypothesize that elevated serum β2‐MG levels in LVH group indicate the presence of an inflammatory response. It has also been reported that serum β2‐MG induced the formation of advanced glycation end products, which have significant cytotoxicity. This leads to an increase in reactive oxygen species (ROS) production, causing cellular dysfunction and inflammatory responses,^32^ which further promote the progression of LVH in T2DM. In T2DM patients, the relationship of serum β2‐MG and LVH may thus influence inflammatory responses and the modification of advanced glycation end products. More detailed potential mechanisms and therapeutic uses require further in‐depth research.

Previous studies have also found that the risk of LVH was influenced by age, sex weight, blood pressure, and renal function.^3^, ^33^ Therefore, to further clarify the relationship between serum β2‐MG and LVH prevalence, subgroup analyses were conducted in different populations. We found that the association of serum β2‐MG with LVH in T2DM patients was consistent among individuals regardless of sex, BMI status, and history of hyperlipidemia. However, the association varied with age, hypertension, and DN status. Stratification led to reduction in sample size that led to a reduction in effect size, which may be one of the reasons for the unstable results. Overall, the consistent direction of all results confirmed the stability and reliability of the core results. Machine learning models were used to analyze the clinical features of LVH, and the importance of β2‐MG for LVH in T2DM was further validated by the resulting prediction model. The threshold serum β2‐MG value was determined as about 1.61 mg/L by a SHAP dependency plot combined with a force diagram. Numerically, thresholds determined by SHAP analysis were close to those obtained by the classical RCS regression analysis. However, further study to determine the serum β2‐MG threshold range is warranted. The above findings supported the relationship between serum β2‐MG levels and LVH in T2DM patients, and confirm its reliability and importance.

The study has several limitations. First, as a retrospective single‐center study, there may have been selection bias in the observational data. However, the impact may have been decreased by the relatively large sample size of our study. Second, as a cross‐sectional study, it could not interpret the causal relationship between serum β2‐MG levels and the occurrence of LVH in T2DM. Nonetheless, we used strict statistical methods to validate the robustness and reliability of the existing findings. Additionally, due to the limitations of the study design and available data, this study may lack some data related to the development of LVH to further correct for confounding factors, such as advanced glycation end products.

## 5 CONCLUSION

In conclusion, this study found that the change in serum β2‐MG levels were an independent influencing factor for LVH in T2DM, suggesting its potential role in the occurrence and development of LVH in T2DM. This finding may contribute to the management of LVH in T2DM patients, thereby strengthening risk assessment and early intervention for individuals at risk of future cardiovascular events and mortality. Finally, further research is needed to elucidate the pathogenesis and potential value of serum β2‐MG in the process of LVH development in T2DM patients.

## AUTHOR CONTRIBUTIONS

Conception or design: Y.L.Z. and X.L. Acquisition, analysis, or interpretation of data: Y.L.Z., G.L.P., W.L.L., Y.L., and H.Y.L. Drafting the work or revising: Y.L.Z., X.L., L.Z., L.C.G., and J.Q.S. Final approval of the manuscript: X.L and M.L.

## FUNDING INFORMATION

This work was supported by the National Natural Science Foundation of China (No. 81873648 to ML) and Chongqing Young and Middle‐aged High‐end Talents Project to M.L.

## DISCLOSURE

No potential conflict of interest relevant to this article was reported.

## Supporting information


**Figure S1.** Flow chart of patient enrollment.
**Table S1.** Baseline clinical characteristics of the study participants according to LVH status.
**Table S2.** Use of anti‐diabetic drugs in the study populationduring hospitalization.
**Table S3.** The correlations between β2‐MG level and various clinical parameters.
**Table S4.** Stratified analysis of the association between β2‐MG and LVH risk in T2DM.

## Data Availability

The datasets used in this study are available from the corresponding authors on reasonable request.
