# Supplementary material for: Association between serum β2‐microglobulin and left ventricular hypertrophy in patients with type 2 diabetes mellitus: A cross‐sectional study
Source: J Diabetes. 2024 Aug 19;16(8):e13599. doi: 10.1111/1753-0407.13599 (PMC11331034; doi:10.1111/1753-0407.13599)
Supplement: Supplementary file 1 — Figure S1. Flow chart of patient enrollment. Table S1. Baseline clinical characteristics of the study participants according to LVH status. Table S2. Use of anti‐diabetic drugs in the study populationduring hospitalization. Table S3. The correlations between β2‐MG level and various clinical parameters. Table S4. Stratified analysis of the association between β2‐MG and LVH risk in T2DM. [file JDB-16-e13599-s001.docx]

**Supplementary**

**
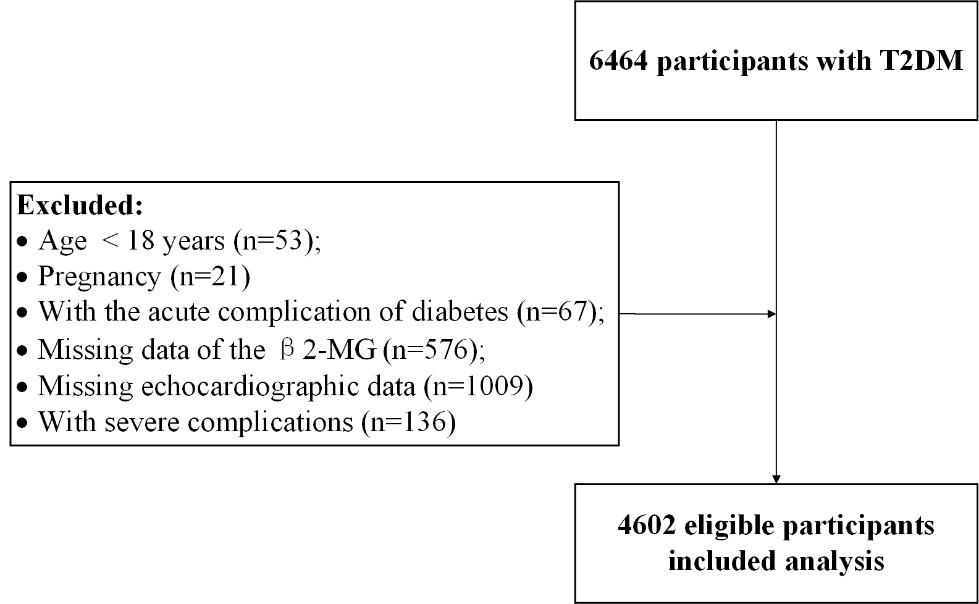
**

**FIGURE S1** Flow chart of patient enrollment

**Table S1** Baseline clinical characteristics of the study participants according to LVH status

| Characteristic | All participants  (n=4602) | Non-LVH group  (n=3170) | LVH group  (n=1432) | *P* value |
| --- | --- | --- | --- | --- |
| β2-MG, mg/L | 1.88 [1.50, 2.53] | 1.78 [1.45, 2.30] | 2.14 [1.66, 3.19] | <0.001 |
| General characteristic |  |  |  |  |
| Age, yr | 59.00 [52.00, 66.00] | 57.00 [50.00, 64.75] | 61.00 [56.00, 68.00] | <0.001 |
| Male sex | 2814 (61.15%) | 2231 (70.38%) | 583 (40.71%) | <0.001 |
| SBP, mmHg | 129.00 [119.00, 140.00] | 127.00 [118.00, 138.00] | 134.00 [122.00, 146.00] | <0.001 |
| DBP, mmHg | 81.00 [75.00, 89.00] | 81.00 [75.00, 88.00] | 81.00 [74.00, 90.00] | 0.226 |
| Smoking | 1894 (41.16%) | 1466 (46.25%) | 428 (29.89%) | <0.001 |
| Drinking | 1793 (38.96%) | 1374 (43.34%) | 419 (29.26%) | <0.001 |
| Height, cm | 163.50 [157.00, 170.00] | 165.00 [159.70, 170.00] | 160.00 [154.00, 166.00] | <0.001 |
| Weight, kg | 65.00 [58.00, 74.00] | 66.50 [58.70, 75.00] | 62.50 [56.00, 70.40] | <0.001 |
| BMI, kg/m^2^ | 24.54 [22.38, 26.73] | 24.45 [22.32, 26.72] | 24.69 [22.49, 26.76] | 0.277 |
| Hypertension | 3035 (65.95) | 1929 (60.85%) | 1106 (77.23%) | <0.001 |
| Hyperlipidaemia | 1157 (25.14%) | 814 (25.68%) | 343 (23.95%) | 0.225 |
| DN | 2110 (45.85%) | 1307 (41.23%) | 803 (56.08%) | <0.001 |
| CHD | 966 (20.99%) | 588 (18.55%) | 378 (26.40%) | <0.001 |
| HF | 93 (2.02%) | 35 (1.10%) | 58 (4.05%) | <0.001 |
| Anti-hypertensive drugs | 860 (18.69%) | 553 (17.44%) | 307 (21.44%) | 0.001 |
| Lipid-lowering drugs | 1140 (24.77%) | 797 (25.14%) | 343 (23.95%) | 0.407 |
| Oral hypoglycemic drugs | 1279 (27.79%) | 908 (28.64%) | 371 (25.91%) | 0.06 |
| Insulin | 1087 (23.62%) | 762 (24.04%) | 325 (22.70%) | 0.34 |
| Laboratory parameters |  |  |  |  |
| FBG, mmol/L | 9.02 [7.29, 11.36] | 9.08 [7.30, 11.44] | 8.92 [7.27, 11.15] | 0.187 |
| HbA1c, % | 8.40 [7.20, 10.00] | 8.40 [7.20, 10.11] | 8.40 [7.20, 9.90] | 0.317 |
| TC, mmol/L | 4.66 [3.96, 5.42] | 4.66 [3.99, 5.39] | 4.64 [3.89, 5.47] | 0.799 |
| LDL, mmol/L | 2.86 [2.39, 3.38] | 2.87 [2.41, 3.36] | 2.85 [2.36, 3.41] | 0.662 |
| TG, mmol/L | 1.72 [1.22, 2.63] | 1.72 [1.21, 2.67] | 1.72 [1.24, 2.51] | 0.673 |
| HDL-C, mmol/L | 1.12 [0.95, 1.31] | 1.11 [0.95, 1.29] | 1.13 [0.95, 1.33] | 0.187 |
| AST, IU/L | 22.13 [18.11, 28.40] | 22.52 [18.40, 29.00] | 21.40 [17.61, 27.18] | <0.001 |
| ALT, IU/L | 22.62 [16.30, 33.02] | 24.00 [16.90, 35.44] | 20.50 [15.04, 29.34] | <0.001 |
| UA, umol/L | 335.41 [277.02, 398.73] | 337.94 [281.00, 399.00] | 329.82 [266.81, 398.00] | 0.011 |
| eGFR, ml/min/1.73m^2^ | 98.75 [81.45, 111.62] | 101.10 [86.73, 113.35] | 92.22 [67.03, 106.54] | <0.001 |
| CRP, mg/L | 3.29 [1.24, 13.66] | 2.90 [1.19, 11.72] | 4.19 [1.40, 18.28] | <0.001 |
| IL6, ng/L | 5.30 [2.00, 14.48] | 4.76 [1.89, 12.92] | 6.81 [2.59, 18.17] | <0.001 |
| NT-proBNP, pg/mL | 66.40 [29.20, 179.32] | 55.30 [24.50, 134.24] | 103.35 [45.20, 399.79] | <0.001 |
| Echocardiographic parameters |  |  |  |  |
| IVST, cm | 1.03 [0.92, 1.15] | 0.98 [0.89, 1.08] | 1.16 [1.05, 1.26] | <0.001 |
| LVDd, cm | 4.60 [4.40, 4.90] | 4.60 [4.30, 4.80] | 4.80 [4.50, 5.00] | <0.001 |
| LVPWT, cm | 0.93 [0.85, 1.02] | 0.90 [0.83, 0.98] | 1.02 [0.94, 1.10] | <0.001 |
| RWT, cm | 0.43 [0.39, 0.47] | 0.41 [0.38, 0.45] | 0.45 [0.41, 0.50] | <0.001 |
| LVM, g | 156.59 [133.43, 184.44] | 144.07 [125.03, 166.64] | 191.79 [163.19, 221.63] | <0.001 |
| LVEF, % | 64.00 [60.00, 67.00] | 64.00 [60.00, 67.00] | 63.00 [60.00, 67.00] | <0.001 |
| E, cm/s | 65.00 [56.00, 77.00] | 65.00 [56.00, 77.00] | 66.00 [55.00, 76.00] | 0.502 |
| A, cm/s | 86.00 [73.00, 98.00] | 84.00 [72.00, 95.00] | 90.00 [77.00, 103.00] | <0.001 |
| E/A ratio | 0.76 [0.64, 0.90] | 0.78 [0.66, 0.93] | 0.72 [0.62, 0.85] | <0.001 |
| Septal e, cm/s | 6.60 [5.90, 7.90] | 6.90 [6.00, 8.00] | 6.00 [5.10, 7.00] | <0.001 |
| Lateral e, cm/s | 9.00 [8.36, 10.00] | 9.21 [8.54, 10.15] | 8.54 [8.00, 9.30] | <0.001 |
| e`, cm/s | 7.80 [7.15, 8.75] | 8.00 [7.32, 9.00] | 7.43 [6.82, 8.15] | <0.001 |

Values are presented as number (%), mean±standard deviation, or median (interquartile range). LVH, left ventricular hypertrophy; SBP, systolic blood pressure; DBP, diastolic blood pressure; BMI, body mass index; DN, diabetic nephropathy; CHD, coronary heart disease; HF, heart failure; FBG, fasting blood glucose; HbA1c, glycated hemoglobin; TC, total cholesterol; TG, triglycerides; LDL-C, low density lipoprotein cholesterol; HDL-C, high density lipoprotein cholesterol; ALT, alanine aminotransferase; AST, aspartate aminotransferase; UA, uric acid; eGFR, estimated glomerular filtration rate; CRP, C-reactive protein; IL-6, interleukin 6; NT-proBNP, N-terminal pro brain natriuretic peptide; β2-MG, β2-microglobulin; IVST, interventricular septal thickness; LVDd, left ventricular end diastolic dimension; LVPWT, left ventricular posterior wall thickness at end-diastole; LVM, left ventricular mass; LVH, left ventricular hypertrophy; LVEF, left ventricular ejection fraction; E, trans-mitral early diastolic peak velocity; A, trans-mitral late diastolic peak velocity; e`= (Septal e+ Lateral e)/2. For non-normally distributed continuous variables were described as median with the interquartile range (25–75%), and the Kruskal-Wallis test was used for comparing multiple subgroups. Categorical variables are represented as frequencies (%) and compared among multiple groups using the χ2 test. P<0.05 represented a statistically significant difference.

**Table S2** Use of anti-diabetic drugs in the study populationduring hospitalization

|  | n (%) |
| --- | --- |
| Insulin | 1087 (23.62) |
| Biguanides | 1135 (24.66) |
| TZDs | 178 (3.87) |
| DPP-4 inhibitors | 627 (13.62) |
| SGLT2 inhibitors | 468 (10.17) |
| α-glucosidase inhibitor | 843 (18.32) |

TZDs, thiazolidinediones; DPP-4, dipeptidyl peptidase-4; SGLT-2, sodium-glucose cotransporter-2 inhibitors.

**Table S3** The correlations between β2-MG level and various clinial parameters

| characteristic | All participants  (n=4603) | |
| --- | --- | --- |
|  | R | *P* value |
| Age, yr | 0.126 | <0.001 |
| SBP, mmHg | 0.132 | <0.001 |
| DBP, mmHg | -0.006 | 0.683 |
| BMI, kg/m2 | 0.019 | 0.194 |
| FBG, mmol/L | -0.012 | 0.435 |
| HbA1c, % | -0.038 | 0.009 |
| TC, mmol/L | -0.037 | 0.012 |
| TG, mmol/L | -0.025 | 0.092 |
| HDL-C, mmol/L | -0.076 | <0.001 |
| LDL-C, mmol/L | -0.018 | 0.231 |
| ALT, IU/L | -0.061 | <0.001 |
| AST, IU/L | -0.004 | 0.769 |
| UA, umol/L | 0.209 | <0.001 |
| eGFR, ml/min/1.73m^2^ | -0.194 | <0.001 |
| NT-proBNP, pg/mL | 0.555 | <0.001 |
| CRP (mg/L | 0.186 | <0.001 |
| IL-6, ng/L | 0.064 | <0.001 |
| Echocardiographic parameters |  |  |
| IVST, cm | 0.137 | <0.001 |
| LVDd, cm | 0.182 | <0.001 |
| LVPWT, cm | 0.168 | <0.001 |
| LVM, g | 0.246 | <0.001 |
| LVEF, % | -0.103 | <0.001 |
| E, cm/s | -0.077 | <0.001 |
| A, cm/s | 0.108 | <0.001 |
| E/A ratio | -0.121 | <0.001 |
| Septal e, cm/s | -0.101 | <0.001 |
| Lateral e, cm/s | -0.113 | <0.001 |
| e`, cm/s | -0.134 | <0.001 |

SBP, systolic blood pressure; DBP, diastolic blood pressure; BMI, body mass index; FBG, fasting blood glucose; HbA1c, glycated hemoglobin; TC, total cholesterol; TG, triglycerides; LDL-C, low density lipoprotein cholesterol; HDL-C, high density lipoprotein cholesterol; AIP, Plasma atherosclerosis index; ALT, alanine aminotransferase; AST, aspartate aminotransferase; UA, uric acid; eGFR, estimated glomerular filtration rate; IVST, interventricular septal thickness; LVDd, left ventricular end diastolic dimension; LVPWT, left ventricular posterior wall thickness at end-diastole; RWT, relative wall thickness; LVM, left ventricular mass; LVEF, left ventricular ejection fraction; E, trans-mitral early diastolic peak velocity; A, trans-mitral late diastolic peak velocity; e`= (Septal e+ Lateral e)/2.

**Table S4** Stratified analysis of the association between β2-MG and LVH risk in T2DM

| β2-MG | OR (95% CIs) | | | | *P*  for trend | *P*  for interaction |
| --- | --- | --- | --- | --- | --- | --- |
|  | Q1 | Q2 | Q3 | Q4 |  |  |
| Age |  |  |  |  |  | **0.005** |
| <60, yr | 1 | 1.20 (0.87, 1.67) | 1.58 (1.15, 2.20) | 2.14 (1.45, 3.14) | <0.001 |  |
| ≥60, yr | 1 | 1.01 (0.77, 1.33) | 1.24 (0.93, 1.65) | 1.32 (0.92, 1.89) | 0.008 |  |
| Sex |  |  |  |  |  | 0.333 |
| Male | 1 | 1.20 (0.87, 1.67) | 1.58 (1.15, 2.20) | 2.14 (1.45, 3.10) | <0.001 |  |
| Female | 1 | 1.45 (1.08, 1.94) | 1.36 (1.00, 1.84) | 1.94 (1.36, 2.70) | <0.001 |  |
| BMI |  |  |  |  |  | 0.638 |
| <25, kg/m^2^ | 1 | 1.29 (0.97, 1.72) | 1.33 (0.98, 1.80) | 2.07 (1.45, 2.98) | <0.001 |  |
| ≥25, kg/m^2^ | 1 | 1.12 (0.81, 1.55) | 1.34 (0.97, 1.86) | 1.54 (1.04, 2.24) | 0.004 |  |
| Hypertension |  |  |  |  |  | **0.012** |
| Yes | 1 | 1.09 (0.86, 1.39) | 1.17 (0.91, 1.52) | 1.49 (1.07, 2.05) | <0.001 |  |
| No | 1 | 1.19 (0.78, 1.84) | 1.41 (0.91, 2.18) | 2.16 (1.35, 3.40) | <0.001 |  |
| Hyperlipidemia |  |  |  |  |  | 0.438 |
| Yes | 1 | 1.04 (0.67, 1.63) | 1.67 (1.06, 2.63) | 2.51 (1.46, 4.33) | <0.001 |  |
| No | 1 | 1.23 (0.96, 1.58) | 1.26 (0.97, 1.63) | 1.68 (1.23, 2.29) | <0.001 |  |
| DN |  |  |  |  |  | **0.012** |
| Yes | 1 | 1.19 (0.89, 1.61) | 1.34 (0.97, 1.85) | 1.81 (1.19, 2.74) | <0.001 |  |
| No | 1 | 0.97 (0.71, 1.31) | 1.19 (0.87, 1.61) | 1.36 (0.99, 1.88) | 0.017 |  |
| CHD |  |  |  |  |  | 0.300 |
| Yes | 1 | 1.24 (0.80, 1.91) | 1.39 (0.88, 2.22) | 1.36 (0.74, 2.48) | 0.010 |  |
| No | 1 | 1.19 (0.93, 1.53) | 1.27 (0.98, 1.63) | 1.81 (1.35, 2.43) | <0.001 |  |

OR, odds ratio; BMI, body mass index; DN, diabetic nephropathy; CHD, coronary heart disease. Adjustment for age, sex, BMI, smoking, drinking, SBP, DBP, hypertension, hyperlipidemia, DN, CHD, HF, antihypertensive drugs, lipid-lowering drugs, anti-diabetes drugs, insulin, FBG, HbA1c, TC, TG, HDL_C, LDL_C, UA, eGFR, ALT, AST, CRP, IL-6, NT-proBNP except the corresponding stratification variable.

Table S5 The test method and equipment of clinical indicators

| Test method | Equipment | Clinical indicators |
| --- | --- | --- |
| Hexokinase method | Automatic biochemical analyzer, Beckmann Kurt Co. LTD, USA | FBG |
| Anion-exchange high-performance liquid chromatography | Automatic hemoglobin A1c analyzer, Beckmann Kurt Co. LTD, USA | HbA1c |
| Colorimetric method | Automatic biochemical analyzer, Beckmann Kurt Co. LTD, USA | TG, TC, HDL-C, LDL-C, ALT, AST, UA, β2-MG, eGFR |
| IMMULITE 1000 Immunoassay System | Chemiluminescence immunoanalyzer, Siemens Healthcare Diagnostics Inc | CRP, IL-6, NT-proBNP |
